# Comparison of Strength and Power Characteristics Before ACL Rupture and at the End of Rehabilitation Before Return to Sport in Professional Soccer Players

**DOI:** 10.1177/19417381231171566

**Published:** 2023-05-19

**Authors:** Luca Maestroni, Anthony Turner, Konstantinos Papadopoulos, Daniel Cohen, Vasileios Sideris, Philip Graham-Smith, Paul Read

**Affiliations:** † ReAct, Bergamo (BG), Italy; ‡ London Sport Institute, School of Science and Technology, Middlesex University, London, UK; § School of Allied Health and Community, University of Worcester, UK; ‖ Masira Research Institute, Faculty of Health Sciences, University of Santander (UDES), Bucaramanga, Colombia; ¶ Mindeporte (Colombian Ministry of Sport) High Performance Centre, Bogota, Colombia; # Aspetar Orthopaedic and Sports Medicine Hospital, Doha, Qatar; ** Aspire Academy, Doha, Qatar; †† Institute of Sport, Exercise and Health, London, UK; ‡‡ Division of Surgery and Interventional Science, University College London, UK; §§ School of Sport and Exercise, University of Gloucestershire, Gloucester, UK

**Keywords:** anterior cruciate ligament, power, reactive strength, soccer, strength

## Abstract

**Background:**

Strength and power is often reduced on the involved versus contralateral limb and healthy controls after anterior cruciate ligament (ACL) reconstruction, but no study has compared with preinjury values at the time of return to sport (RTS).

**Hypothesis:**

Divergent recovery patterns in strength and power characteristics will be present at RTS relative to preinjury baseline data and healthy matched controls.

**Study Design:**

Cohort study.

**Level of Evidence:**

Level 3.

**Methods:**

Isokinetic strength tests, bilateral and single-leg countermovement jumps (CMJ; SLCMJ) were measured before ACL rupture in 20 professional soccer players. These then had surgical reconstruction (ACL group) and completed follow-up testing before RTS. Healthy controls (uninjured group) were tested at the same time as the ACL group preinjury. Values recorded at RTS of the ACL group were compared with preinjury. We also compared the uninjured and ACL groups at baseline and RTS.

**Results:**

Compared with preinjury, ACL normalized quadriceps peak torque of the involved limb (difference = -7%), SLCMJ height (difference = -12.08%), and Reactive Strength Index modified (RSImod) (difference = -5.04%) were reduced after ACL reconstruction. No significant reductions in CMJ height, RSImod, and relative peak power were indicated at RTS in the ACL group when compared with preinjury values, but deficits were present relative to controls. The uninvolved limb improved quadriceps (difference = 9.34%) and hamstring strength (difference = 7.36%) from preinjury to RTS. No significant differences from baseline were shown in SLCMJ height, power, and reactive strength of the uninvolved limb after ACL reconstruction.

**Conclusion:**

Strength and power in professional soccer players at RTS after ACL reconstruction were often reduced compared with preinjury values and matched healthy controls.

**Clinical Relevance:**

Deficits were more apparent in the SLCMJ, suggesting that dynamic and multijoint unilateral force production is an important component of rehabilitation. Use of the uninvolved limb and normative data to determine recovery may not always be appropriate.

Anterior cruciate ligament (ACL) injuries in elite soccer players incur a high burden,^2^ with substantial time-loss and economic cost.^10^ This traumatic event often results in surgical reconstruction, and return-to-sport (RTS) time is on average ~8 months.^37^ Although most (83%) elite athletes return to their preinjury level of competition after ACL reconstruction,^22^ this is often accompanied by an increased risk of ipsilateral and contralateral injury,^17,18^ early onset of posttraumatic osteoarthritis, and sports performance deterioration.^8,22-24^

Strength and power are reduced after ACL reconstruction.^29^ Strength assessment has commonly included isokinetic testing of knee extension and flexion peak torque, with established excellent reliability scores documented.^1,14,38^ Deficits in peak knee extension and flexion torque are commonly displayed in the ACL reconstructed limb compared with the uninvolved side and healthy controls after rehabilitation at the time of RTS.^15,29^ In addition, jump performance is often used to quantify dynamic multijoint force production and can discriminate rehabilitation status.^31,32^ Countermovement jump (CMJ) performance variables can help practitioners to quantify neuromuscular qualities that underpin movements inherent to soccer such as sprinting, jumping, and change of direction.^13^ However, it has been suggested that single-leg dynamic tasks are more representative of limb strength due to their higher relative force demands,^7^ whereas bilateral jumping and landing tasks occur at a higher velocity. Furthermore, compensation strategies are restricted to interjoint in unilateral movements, whereas bilateral jumping can provide more options to unload the ACL reconstructed limb via both interjoint and interlimb.^28^ The differing demands of the bilateral and unilateral tasks may reveal specific deficits, warranting the inclusion of both in the assessment of neuromuscular performance for athletes during rehabilitation aiming to return to a high level of competition.

Research assessing strength and power characteristics in athletes after ACL reconstruction has been limited mostly to cross-sectional studies at single timepoints or around the time of RTS.^16-20,31,32,34,35^ Residual deficits in vertical jump height, lower limb power, and reactive strength appear to be present after ACL reconstruction.^27,32,34^ Lower quadriceps strength and reduced plyometric ability have also displayed associations with increased risk of contralateral reinjury.^17,18^ However, the available research has used the contralateral limb or values from matched controls to determine whether deficits are present. There is potential for deterioration of the uninvolved contralateral limb after surgery due to deconditioning/lack of exposure.^44^ Without preinjury baseline physical characteristics, it is impossible to determine whether athletes have returned to previous strength and jump performance values. It is also unknown whether matched controls provide an accurate representation of baseline/preinjury performance. A prospective study monitoring strength and power qualities from tests that are commonly used as part of RTS assessment in elite soccer players before and after ACL rupture and reconstruction may help guide performance recovery and determine the accuracy of proxy measures, including the uninvolved limb and comparison values of healthy controls.

Our aim was to examine the changes in strength and power performance after completion of rehabilitation at the time of RTS compared with preinjury baseline data and compared with healthy matched controls. Using these data, we examined how preinjury benchmark data can be used to guide performance recovery and inform physical readiness as part of RTS decision-making. Our specific research questions included (1) to what extent performance metrics are recovered at the time of RTS after ACL reconstruction and (2) how accurate is the use of the contralateral limb and group/control normative data as proxy measures for determining performance recovery when preinjury data exist.

## Methods

### Participants

A total of 20 soccer players (24.7 ± 3.4 years; height, 175.3 ± 7.0 cm; weight, 69.5 ± 10.7 kg) participating in the Qatar Stars and Gas Leagues attended a periodic health evaluation between 2017 and 2019, and subsequently went on to sustain an ACL rupture before undergoing ACL reconstruction (ACL group). The majority of ACL grafts were bone-patella-tendon bone (80%), with the remaining players (20%) all semitendinosus and gracilis hamstring tendon grafts. Only participants with no history of previous ACL injury/surgery, or other knee ligament or cartilage injury/surgery of either the operated or nonoperated leg at the time of the periodic health evaluation were included. All athletes were treated at the same Orthopaedic and Sports Medicine Hospital. Rehabilitation was delivered 5 days per week and divided into early, intermediate, and advanced phases. The focus of the early phase was on controlling swelling, restoring range of motion, and activation of the knee extensor and flexor muscles. The goal of the intermediate and advanced phases were to optimize muscle strength, proprioception, and neuromuscular control, and complete a phased running progression program. On completion of these phases, players took part in an onfield sports-specific training and conditioning block.

We also recruited 35 (uninjured) controls (23.8 ± 2.8 years; height, 173.8 ± 5.4 cm; weight, 71.6 ± 6.3 kg) from the same leagues who attended preseason screening at the national sports medicine institution and were selected randomly from a pool of 300 athletes. Inclusion was based on having no history of ACL injury and being free from any severe injury (defined as >28 days time-loss) in the previous 12 months, verified via a national injury audit. Clubs competing in the stated leagues within Qatar regularly complete formalized strength and conditioning including resistance training, speed, agility, and plyometrics. Before participating, all participants provided informed written consent and ethical approval was provided (Institutional Review Board: F2017000227).

### Experimental Approach to the Problem

To address our stated aims, we separated the study into 4 components. In part 1, we compared strength and power characteristics of the ACL group with those of the uninjured group using both the preinjury (baseline) data and performance after the completion of rehabilitation of the ACL group. Preinjury baseline data are not commonly available, forcing clinicians to instead use either peers/published data and or the contralateral limb as proxy benchmarks after ACL reconstruction,^29^ but the former has not been explored. In part 2, we monitored the trajectory of strength and power performance of the uninvolved limb in the ACL group by comparing isokinetic and SLCMJ assessment scores at 2 time points: preinjury and at the end of rehabilitation before RTS. Conflicting evidence is available about the detrimental effect of ACL reconstruction and subsequent deconditioning on the uninvolved limb.^26,36,44^ Currently, no study has conducted an assessment of strength and power characteristics of the uninvolved limb before and after ACL reconstruction after structured full-time rehabilitation. In part 3, we measured the effect of ACL reconstruction and rehabilitation on the injured limb by comparing isokinetic and SLCMJ performance scores at 2 timepoints: preinjury and at the end of rehabilitation, after sports-specific reconditioning prior to RTS. Finally, in part 4, we investigated the effect of ACL reconstruction on bilateral CMJ performance by comparing preinjury and RTS values.

### Procedures

**Figure 1.**
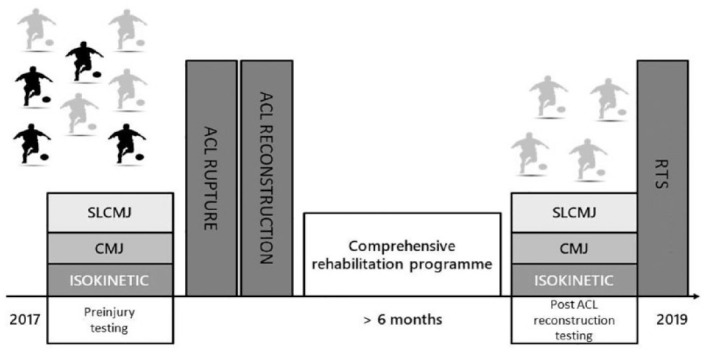
Schematic representation of the study design. Uninjured and injured players are depicted in black and gray, respectively. ACL, anterior cruciate ligament; RTS, return to sports.

A schematic diagram of our study is represented in Figure 1. A test battery consisting of isokinetic strength assessment, CMJ, and SLCMJ was performed. The ACL reconstructed cohort was screened 33.9 ± 29.6 weeks before the ACL rupture, and assessed at the end of rehabilitation before RTS (30.3 ± 7.2 weeks postsurgery). Players completed a standardized warm up consisting of 5 minutes on a cycle ergometer, bilateral and unilateral bodyweight squats, and bilateral CMJs at 50%, 75%, and 100% maximum effort.^33^ Test conditions and procedures were replicated at each assessment.

### Isokinetic Knee Extension and Flexion Strength

Maximal quadriceps knee extension peak torque (Quad PT Rel) and hamstring flexion peak torque (HS PT Rel) relative to body mass (Nm/kg) were measured using an isokinetic dynamometer (Biodex Medical Systems). Players were in a seated position with the hip flexed to 90°. Five repetitions of concentric knee extension and flexion were performed at 60 deg/s with the highest peak torque value recorded.^42^ Peak torque values were reported as a percentage of the person’s body mass. Procedures were explained to participants after which they completed 3 practice repetitions. Testing then commenced after 60 s. Limb order was randomized. The dominant limb of healthy controls was defined as the preferred kicking leg. Standardized, vigorous verbal encouragement was provided throughout. Each participant had previous experience of isokinetic testing and all tests were conducted by the same physiotherapist with >5 years experience in the relevant test procedures.

### CMJ (Bilateral/Single)

Participants were instructed to stand fully upright, hands on hips, and align their feet on a synchronized dual force plate system (ForceDecks Version 1.2.6109, Vald Performance). Before initiation of the test, each player was instructed to remain motionless for a minimum of 3 s to ensure a stable baseline of force at bodyweight was obtained. Players then performed a downward motion (descent phase) until they reached their preferred self-selected depth, before rapidly reversing the motion by triple extending at the hip, knee, and ankle. The aim of the task was to achieve their maximal vertical displacement of the center of mass. Hands remained on hips throughout and no bending of the knees was permitted while airborne. The procedures were replicated for the SLCMJ, except the nontest leg was positioned with the hip and knee at 90º and no obvious swinging was allowed to minimize contralateral propulsion. Limb order was randomized. Two trials were performed with a 30 s rest period between each jump, with the best trial recorded for statistical analysis.

All data were recorded at a sampling rate of 1000 Hz. The initiation of the jump was defined by a 20 N change from bodyweight calculated during the quiet standing period and the instant of take-off, when the total vertical force dropped below 20 N. We selected 3 outputs, which are commonly reported in jump performing testing of healthy athletes and which can also be estimated using other lower cost technologies than force platform. Jump height was calculated from the impulse-momentum relationship derived take-off velocity and equation of constant acceleration (velocity at take-off squared divided by 2 × 9.81 (*v*^2^/2*g*)). Peak power was measured and normalized to bodyweight W/kg (Peak Power Rel) during the propulsion phase. Reactive strength index modified (RSImod) was calculated by dividing jump height by contraction time (determined from movement onset to time to take-off).^39^

Intraday reliability analysis was conducted on baseline preinjury scores of the ACL group. The between trial reliability was analyzed using a 2-way random effects intraclass correlation coefficient [ICC (2,1)] with 95% CI.^21^ The ICCs were analyzed as single measures. Coefficient of variation (CV%), 95% CI, and standard error of measurement (SEM) were also calculated. Reliability scores were categorized as acceptable if the CV was ≤10%,^40^ and were further categorized as “excellent” if ICC was >0.90, “good” between 0.75 and 0.90, “moderate” between 0.50 and 0.75, and “poor” <0.50.^21^

CMJ height, relative peak power, and reactive strength displayed “excellent” reliability, with ICC ranging from 0.945 to 0.978, and CV between 2.1% and 8.6% (Appendix Table A1, available in the online version of this article). SLCMJ height, RSImod, and jump height symmetry displayed “excellent” reliability, with ICCs ranging from 0.901 to 0.960 and CV between 4.2 and 5.9 (Appendix Table A1, available online). Relative peak power showed CV <10%, and ICC between 0.781 and 0.860.

### Statistical Analysis

The distribution of the data was checked using the Shapiro-Wilk normality test. Descriptive statistics (means and standard deviations) for all variables were calculated. Percentage changes from preinjury to post-ACL-reconstruction were calculated for each player using the percentage difference and then averaged.

In part 1, an independent samples *t* test or Mann-Whitney U test were used to examine differences in anthropometrics and physical performance variables between the ACL and uninjured groups.

For parts 2 to 4, paired-samples tests or Wilcoxon rank sum test were used to detect statistical differences between preinjury and postsurgery physical performance variables. The 2-way repeated measures ANOVA was used to examine the influence and interaction of time and/or injury (performance on the injured limb) for each test variable in the ACL group.

In all parts, Bonferroni correction was applied to reduce the risk of type I error with multiple statistical tests (adjusted α = 0.025 and α = 0.017 for isokinetic dynamometry and dual force plate system derived variables, respectively). Hedges *g* effect sizes (ES) with 95% CIs were calculated to interpret the magnitude of these differences with the following classifications: standardized mean differences of 0.2, 0.5, and 0.8 for small, moderate, and large ES, respectively.^41^ Significance was set at *P* < 0.05. Data processing and descriptive statistics were processed using SPSS (Version 25).

## Results

### Part 1: Strength and Power Characteristics of ACL Reconstructed Group Versus Healthy Matched Controls

Baseline (preinjury) anthropometric, strength, and power characteristics of the ACL reconstructed group were not significantly different form healthy matched controls (Table 1).

**Table 1.** Isokinetic, SLCMJ, and bilateral CMJ results of each group

Test	Group 1 Preinjury (n = 20)		Group 2 Healthy Controls (n = 35)	Preinjury vs Controls Effect Size (95% CI)	Preinjury vs Controls *P* value
	Involved Limb	Uninvolved Limb	Dominant Limb		
Quad PT Rel, Nm/kg	3.2 ± 0.37	3.13 ± 0.44	3.06 ± 0.4	0.35 (-0.21 to 0.92)	0.20
HS PT Rel, Nm/kg	1.75 ± 0.26	1.79 ± 0.3	1.68 ± 0.22	0.29 (-0.27 to 0.86)	0.34
SLCMJ height, cm	18.5 ± 4.4	19.2.2 ± 3.4	18.8 ± 2.3	-0.09 (-0.65 to 0.47)	0.79
SLCMJ RSImod	0.22 ± 0.08	0.24 ± 0.07	0.24 ± 0.05	-0.25 (-0.82 to 0.31)	0.51
SLCMJ peak power rel, W/kg	31.7 ± 4.3	32.7 ± 4.4	31.9 ± 4.2	-0.05 (-0.61 to 0.52)	0.86
CMJ height, cm	36.4 ± 7.4		37.5 ± 3.6	-0.22 (-0.78 to 0.35)	0.23
CMJ RSImod	0.46 ± 0.11		0.49 ± 0.07	-0.30 (-0.86 to 0.27)	0.35
CMJ peak power rel, W/kg	52.1 ± 6.3		52.8 ± 4.9	-0.13 (-0.69 to 0.44)	0.70

CMJ, countermovement jump; HS PT Rel, hamstring flexion peak torque relative to body mass; Quad PT Rel PT, quadriceps knee extension peak torque relative to body mass; RSImod, Reactive Strength Index modified; SLCMJ, single-leg CMJ.

Normalized Quad and HS PT were significantly higher in the uninvolved limb of the ACL group before RTS compared with those who were uninjured (*g* = 0.77; 95% CI [0.19, 1.36]; *P* = 0.02, and *g* = 0.77, 95% CI [0.19, 1.35]; *P* < 0.01, respectively). There were no significant differences in SLCMJ height, RSImod, or relative peak power between the uninvolved limb of the ACL group and uninjured controls (Appendix Table A2).

Normalized HS PT was significantly higher in the reconstructed limb of the ACL group after rehabilitation compared with uninjured controls (*g* = 1.32, 95% CI [0.70, 1.93]; *p* < 0.01), whereas there were no significant between-group differences in normalized Quad PT (Appendix Table A3).

There were large significant differences between the ACL group after surgery and uninjured controls in SLCMJ height (*g* = -1.64, 95% CI [-2.28, -0.99]; *P* < 0.01), RSImod (*g* = -0.93, 95% CI [-1.52, -0.34]; *P* < 0.01), and jump height symmetry (*g* = -1.51, 95% CI [-2.14, -0.87]; *P* < 0.01) (Appendix Table A3).

There were large significant differences between the ACL group after surgery and uninjured controls in CMJ height (*g* = -1.17, 95% CI [-1.77, -0.56]; *P* < 0.01) and RSImod (*g* = -0.89, 95% CI [-1.48, -0.30]; *P* < 0.01). Moderate differences in relative peak power (*g* = -0.76, 95% CI [-1.34, -0.18]; *P* < 0.01) were also present between groups (Table 2).

**Table 2.** CMJ test results of each group

Test	Group 1 Preinjury (n = 20)	Group 1 Postinjury(n = 20)	Pre vs Post effect size (95% CI)	Pre vs Post P value	Pre-Post Percentage difference (95%CI)	Group 2: Healthy Controls (n = 35)	Postinjury vs controls effect size (95%CI)	Postinjury vs controls *P* value
CMJ height, cm	36.4 ± 7.4	33.2 ± 3.7	0.54 (-0.12 to 1.19)	0.04	-5.92% (-7.76 to -4.08)	37.5 ± 3.6	-1.17 (-1.77 to -0.56)	<0.01
CMJ RSImod	0.46 ± 0.11	0.42 ± 0.09	0.39 (-0.26 to 1.04)	0.08	-5.51% (-7.22 to -3.8)	0.49 ± 0.07	-0.89 (-1.48 to -0.30)	<0.01
CMJ peak power rel, W/kg	52.1 ± 6.3	49.1 ± 4.6	0.53 (-0.12 to 1.19)	0.04	-4.94% (-6.47 to -3.41)	52.8 ± 4.9	-0.76 1.34 to -0.18)	<0.01

CMJ, Countermovement jump; RSImod, Reactive Strength Index modified.

### Part 2: Effect of ACL Reconstruction on Uninjured Limb

Uninvolved limb preinjury and post ACLR performance for each of the participants is shown online in Appendix Figures A1b, A2b, and A3b). There was no significant main effect of time (F(1,19) = 0.43; *P* = 0.84), but there was a significant main effect of injury on normalized Quad PT (F(1,19) = 7.996, *P* = 0.01). A significant interaction effect between time and injury was present (F(1,19) = 32.8, *P* < 0.01), showing an increase in normalized Quad PT in the uninvolved limb. No main effect of injury was observed for normalized HS PT (F(1,19 ) = 0.47; *P* = 0.5) and no significant interaction effect between time and injury (F(1,19) = 3.8; *P* = 0.07). There was a significant main effect of time only on normalized HS PT (F(1,19) = 7.35; *P* = 0.01), which showed improvements in normalized HS PT in the uninvolved limb attributable to the passage of time only after surgery.

There were no significant main or interaction effects of time and/or injury on SLCMJ height, relative peak power, and RSImod in the uninvolved limb.

Moderate ES differences in normalized Quad PT were observed post ACL reconstruction in comparison with preinjury values (*g* = 0.57, 95% CI [-0.08, 1.23]; *P* = 0.02), whereas there were no significant differences in normalized HS PT (Appendix Table A2).

### Part 3: Effect of ACL Reconstruction on the Injured Limb

Involved limb preinjury and post ACLR performance for each of the participants is shown online in Appendix Figures A1a, A2a, and A3a. There was no significant main effect of time (F(1,19) = 0.43; *P* = 0.84), but there was a significant main effect of injury on normalized Quad PT (F(1,19) = 7.996; *P* = 0.01). A significant interaction effect between time and injury was present (F(1,19) = 32.8; *P* < 0.01), showing deterioration in normalized Quad PT in the ACL reconstructed limb. No main effect of injury was observed for normalized HS PT (F(1,19) = 0.47; *P* = 0.5) and there was no significant interaction effect between time and injury (F(1,19) = 3.8; *P* = 0.07). A significant main effect of time on normalized HS PT (F(1,19) = 7.35, *P* = 0.01) was shown, which indicates improvements in normalized HS PT in the ACL reconstructed limb after surgery.

There was a significant main effect of time (F(1,19) = 5.28, *P* = 0.03) and injury (F(1,19) = 49.56; *P* < 0.01) on SLCMJ height, relative peak power (F(1,19) = 31.75; *P* < 0.01), and RSImod (F(1,19) = 45.42; *P* < 0.01) in the ACL reconstructed limb. A significant interaction effect was present between time and injury in jump height (F(1,19) = 11.53; *P* < 0.01), relative peak power (F(1,19) = 5.86; *P* = 0.03), and RSImod (F(1,19) = 8.02; *P* = 0.01), indicating SLCMJ performance had not returned to baseline. Conversely, normalized HS PT was significantly higher after ACL reconstruction compared with preinjury values (*g* = 0.90, 95% CI [0.23, 1.58]; *P* < 0.01). No significant differences in normalized Quad PT were present (Appendix Table A3).

### Part 4: Effect of ACL Reconstruction on CMJ Performance

Preinjury and post ACLR CMJ height for each of the participants is shown in Online Appendix Figure A4. No significant reductions in CMJ RSImod were present between the ACL reconstructed group before ACL rupture and after reconstruction at the time of RTS. Although not achieving our determined alpha level, moderate differences in CMJ height (*g* = 0.54, 95% CI [-0.12, 1.19]; *P* = 0.04) and relative peak power (*g* = 0.53, 95% CI [-0.12, 1.19]; *P* = 0.04) were present between the ACL reconstructed group before injury and after reconstruction at the end of rehabilitation around at the time of RTS (Table 2).

## Discussion

Our aim was to examine how preinjury data can be used to guide performance recovery and inform physical readiness as part of RTS decision-making. Cumulatively, the results indicate that residual deficits in strength and power are present after ACL reconstruction (7.6 ± 1.8 months postsurgery) and the pattern of recovery is diverse across tests and metrics selected. Use of both the uninvolved limb and normative data of matched controls as a proxy measure to determine the level of performance recovery may not always be appropriate to estimate the degree of recovery, and practitioners are encouraged to collect routine preinjury data where possible to most accurately assess physical readiness to RTS.

### Recovery of Involved Limb and Bilateral Performance

Deficits in knee extension peak torque relative to controls have been documented in male multidirectional team sport athletes >6 months after surgery.^29^ In our study, group mean values indicated normalized quadriceps strength levels in the ACL cohort at the time of RTS were in line with recommended thresholds (>3.0 Nm/kg at 60 deg/s),^43^ and did not significantly differ from the uninjured group, indicating this should be the first rehabilitation target. However, there was some variability across participants (Online Appendix Figure A2a), and normalized quadriceps strength of the involved limb post ACL reconstruction showed reduced values compared with those recorded preinjury (*g* = -0.48; *P* = 0.04), suggesting that comparison with preinjury values may add important information regarding strength recovery after ACL reconstruction. Our professional athletes completed a progressive strength training intervention during rehabilitation, which has been shown to attenuate strength deficits after ACL rehabilitation.^43^ However, normalized quadriceps strength on the involved limb was reduced compared with baseline values and substantially lower than the contralateral limb at the end of rehabilitation. These data indicate that both individual limb torque scores need to be considered in RTS decision-making, and when preinjury data are available, assessment of symmetry may be secondary compared with attainment of the athletes own benchmark scores on each limb. Longer rehabilitation periods (≥9 months) may also be needed to recover knee extensor torque deficits,^3^ Optimal knee extension strength recovery is associated with reduced risk of future knee injury and osteoarthritis,^9,12^ greater subjective knee functional scores (IKDC),^6^ articular cartilage status,^11^ and reduced interlimb and intralimb maladaptive compensation strategies during unilateral and bilateral jumping and landing tasks.^28^ Targeted interventions with a maximal strength emphasis should be integral components of rehabilitation until, at the very least, normative values (>3.0 Nm/kg) are met.

**Figure 2.**
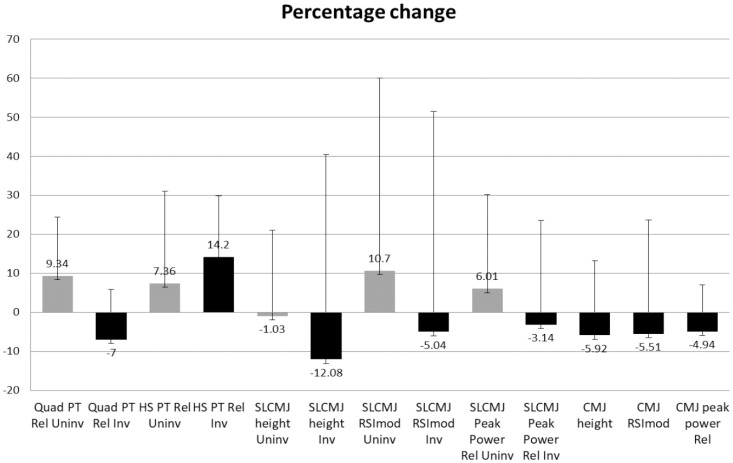
Percentage changes from preinjury to post ACL reconstruction of all variables analyzed. ACL, anterior cruciate ligament; CMJ, countermovement jump; HS PT Rel, hamstring peak torque relative to bodyweight; Inv, involved; peak power Rel, relative peak power; Quad PT Rel, quadriceps peak torque relative to bodyweight; RSImod, reactive strength index modified; SLCMJ, single-leg CMJ; Uninv, uninvolved.

Our study revealed a significant reduction in CMJ height, RSImod, and relative peak power in ACL reconstructed players in comparison with baseline preinjury performance (CMJ height *g* = -0.54, *P* = 0.04; RSImod *g* = -0.39, *P* = 0.08; relative peak power *g* = -0.53, *P* = 0.04) and healthy controls (CMJ height *g* = -1.17, *P* < 0.01; RSImod *g* = -0.89, *P* < 0.01; relative peak power *g* = -0.76, *P* = 0.01). For some players, CMJ height was substantially lower than their preinjury baseline (Figure A4). Other researchers have suggested that recovery of CMJ height is still incomplete at the time to RTS in comparison with healthy controls.^35^ There was also evidence of large reductions in SLCMJ height (*g* = -1.64, *P* < 0.01) and RSImod (*g* = -0.93, *P* < 0.01) on the involved limb, and this trend was consistent across most participants (Appendix Figure A1, available online). To execute a single-leg jump, there is a higher relative force requirement compared with bilateral (estimated ~1.62 times of those in a CMJ) to displace body mass vertically, resulting in slower movement velocities.^7^ We observed a greater reduction in SLCMJ height (-12.08%), than in CMJ height (-5.92%) after ACL reconstruction (Figure 2). Therefore, as the deficits in SLCMJ height were twice the magnitude of those in the CMJ, it could be suggested that SLCMJ height offers a better reflection of limb capacity compared with measurement of the same variable in a bilateral jump. The CMJ task allows athletes to redistribute their impulse production via interlimb compensations in an attempt to maintain similar jump heights.^35^ These data can be derived from dual force platforms but such technology is not commonly available to clinicians. Measurement of SLCMJ height is obtainable using a variety measurement tools and may be a useful indicator to determine the recovery of limb capacity around the time of RTS.

Previous research has reported SLCMJ normative scores of >17 cm in multidirectional field sport athletes at the late stages of rehabilitation.^32^ These values are in line with the results of our study (Online Appendix Figure A5), which included healthy professional soccer players. Therefore, ~18 cm may represent a realistic target to achieve by the end of rehabilitation for field sport athletes if preinjury values are not available. However, as many athletes baseline scores were higher (Appendix Figure A1a), this further highlights the importance of routine preinjury data collection at regular intervals to ensure the most accurate benchmark is established. In addition, the ACL reconstructed limb showed reduced RSImod in comparison with the dominant limb of healthy controls (Appendix Figure A5). Decreased stretch shortening cycle performance has been documented recently in similar cohorts,^19,27,34^ and is associated with higher risk of ipsilateral and contralateral ACL injury,^17,18^ as well as reduced sports performance.^25,30^ Thus, increased emphasis on reconditioning strategies to recover ballistic performance needs to be embedded in the RTS pathway, together with progressive strength training interventions.^4,5^

### Use of Proxy Measures in Decision-Making

When making RTS decisions, comparison with preinjury is often impracticable. Our data suggest that, in single-leg jumping tasks, healthy matched controls including mean values for team mates or published data for a similar playing level could provide a suitable reference of the minimum target that should be achieved in monitoring the recovery of physical performance after ACL reconstruction. However, utilization of strength scores in healthy controls may not follow the same pattern. Overestimation of functional improvements during rehabilitation have been reported previously when using preoperative scores on the contralateral limb as a reference value at the time of RTS owing to a bilateral reduction in physical performance after ACL reconstruction inflating limb symmetry indexes.^44^ In contrast, we observed that normalized quadriceps and hamstring strength improved from preinjury after the completion of rehabilitation on the uninvolved limb in the ACL reconstructed group and scores were greater than matched controls (Appendix Figure A5), suggesting an underestimation in the degree of recovery if the latter comparison was used. Conversely, involved limb reductions in quadriceps strength at the time of RTS were greater when compared with preinjury data (7%) and healthy controls (2.6%), suggesting use of healthy control values would overestimate the degree of recovery for involved limb quadriceps strength. If the contralateral limb was used postinjury, a larger (14%) between-limb difference was present, and this would underestimate the degree of recovery. Our participants were full-time athletes attending rehabilitation 5 days per week, of which rehabilitation knee extension and flexion strength were considered a priority. This suggests that when a comprehensive rehabilitation program including progressive strength training is followed, comparison with matched controls alone is not enough, although it does represent the first achievable milestone to ensure strength recovery. However, it should be considered that training age and routine exposure to strength and conditioning of the healthy controls were not examined. Similarly, use of the contralateral limb may be misleading and can underestimate recovery when significant training adaptations have occurred. Thus, proxy measures to determine the level of performance recovery may not always be appropriate.

Large performance reductions were observed in bilateral CMJ height and RSImod based on healthy controls values, but the corresponding deficits based on true benchmark values were classified as moderate, suggesting a potential underestimation of recovery of these metrics when using healthy control data. SLCMJ performance on the uninvolved limb showed no significant difference preinjury versus RTS, although there was a slight reduction in jump height. Our data indicate that both healthy controls and the unaffected limb could be used as a references in monitoring SLCMJ performance recovery (ie, achievement of preinjury baseline values) on a group level, but caution should be applied as several athletes preinjury SLCMJ scores were greater than these values.

Our data also suggests that a comprehensive rehabilitation program can mitigate reductions in contralateral knee strength and power secondary to surgery and reduced load exposure. Maintaining or even increasing quadriceps and plyometric qualities can have important implications in reducing subsequent ACL injury risk to the uninjured limb in male athletes after ACL reconstruction,^18^ and thus should be monitored during rehabilitation. Further research is encouraged to measure temporal recovery across multiple timepoints in these physical qualities to more accurately determine the trajectory of recovery.

### Limitations

Changes from baseline preinjury scores after ACL reconstruction should be interpreted relative to the measurement error in the metrics used (Appendix Table A1). CMJ height and relative peak power displayed CV values of 2.7% and 2.1%, respectively. The corresponding changes after ACL reconstruction and rehabilitation were 5.92% and 4.94%, indicating that a “real” change had occurred with differences larger than the observed measurement error. RSImod reduced by 5.51%, but the CV value was 8.6%, which suggests the observed differences were within the error range and could be considered less meaningful. Similarly, only SLCMJ height showed changes after ACL reconstruction larger than the measurement error (-12% reduction; CV, 5.2%), whereas RSImod and relative peak power had a greater CV% relative to the observed percentage change. In addition, we were not able to collect follow-up data on the uninjured controls to determine what is “normal” seasonal variation in these metrics.

Our sample size precluded us from conducting analysis based on graft type and this may have an effect on strength and power qualities. The majority of our players had a bone-patellar tendon-bone graft, which can explain the incomplete and delayed recovery of knee extensor and concentric jump outputs deficits, in comparison with similar cohorts with a semitendinosus/gracilis graft type.^31^ Finally, none of the assessments directly assessed eccentric qualities, which may show divergent recovery patterns and deficits, and therefore our conclusions should be considered to be related principally to concentric strength/jump outputs that ultimately reflect capacity to generate concentric impulse. Our data were limited to adult male professional football players. Therefore, generalization of these results to pediatric, adolescent, and female athletes requires caution. Although the surgeons and rehabilitation specialists involved belonged to the same Orthopaedic and Sports Medicine Hospital, potential variations in surgical techniques and rehabilitation strategies could have been present and should also be acknowledged.

## Conclusion

The current study indicates that ACL reconstruction has a detrimental effect on strength and power characteristics in professional soccer players but the pattern was diverse. Peak knee extension strength, CMJ and SLCMJ height, RSImod, and relative peak power values at the end of rehabilitation before RTS remained below those recorded preinjury. Furthermore, in spite of the fact that players approached strength values deemed sufficient in the ACL reconstructed limb and exceeded these criteria in the contralateral limb, large differences in SLCMJ height and RSImod were still evident on the ACL reconstructed limb in comparison with uninjured matched controls. These differences were smaller when assessed bilaterally (ie, CMJ test), indicating that SLCMJ can be used to more closely evaluate the recovery of individual limb physical capacity. These data can be easily obtained using a variety of cost effective methods, especially compared with isokinetic assessments, which require expensive equipment and are time-inefficient.

Our findings are summarized in Table 3, and have clinical implications to help guide the RTS process. Cumulatively, we suggest that an optimal approach to determine physical recovery at the time of RTS would include the following: (1) data collected as early as possible (baseline preinjury if available or, if not, preoperative values on the uninvolved limb) to inform readiness to RTS as this should be considered the gold standard reducing the need for proxy measures of limb recovery, which can overestimate or underestimate limb function; (2) consider both absolute scores on each limb and not just symmetry values; (3) in situations where baseline preinjury data are not available, compare with uninjured matched controls to ensure minimum standards are met. In addition, we suggest to include both unilateral and bilateral assessments with a range of demands across the strength, power, and velocity spectrum to ensure performance is measured under different task constraints.

**Table 3.** Summary table

Research Question	Significant Findings
Do the strength and power characteristics differ in soccer players who sustained an ACL injury and underwent subsequent reconstructive surgery to those of uninjured players?	No difference between groups in strength, power and reactive strength characteristics at baseline assessment, but lower performance was indicated in ACL reconstructed players at the end of rehabilitation
How does ACL reconstruction effect isokinetic knee extension/flexion strength and SLCMJ performance on the uninvolved limb?	Increase in quadriceps and hamstring strength from preinjury to RTS. No significant differences from preinjury in SLCMJ height, power, and reactive strength after ACL reconstruction
How does ACL reconstruction effect isokinetic knee extension/flexion strength and SLCMJ performance on the involved limb?	Increase in hamstring strength from preinjury to RTS Decrease in quadriceps strength, SLCMJ height and reactive strength after ACL reconstruction
How does ACL reconstruction effect CMJ performance?	Decrease in jump height, reactive strength, and power after ACL reconstruction

ACL, anterior cruciate ligament; CMJ, countermovement jump; RTS, return to sport; SLCMJ, single-leg CMJ.

## Supplemental Material

sj-docx-1-sph-10.1177_19417381231171566 – Supplemental material for Comparison of Strength and Power Characteristics Before ACL Rupture and at the End of Rehabilitation Before Return to Sport in Professional Soccer Players Click here for additional data file. Supplemental material, sj-docx-1-sph-10.1177_19417381231171566 for Comparison of Strength and Power Characteristics Before ACL Rupture and at the End of Rehabilitation Before Return to Sport in Professional Soccer Players by Luca Maestroni, Anthony Turner, Konstantinos Papadopoulos, Daniel Cohen, Vasileios Sideris, Philip Graham-Smith and Paul Read in Sports Health
