# Supplementary material for: Comparison of Strength and Power Characteristics Before ACL Rupture and at the End of Rehabilitation Before Return to Sport in Professional Soccer Players
Source: Sports Health. 2023 May 19;15(6):814–23. doi: 10.1177/19417381231171566 (PMC10606975; doi:10.1177/19417381231171566)
Supplement: sj-docx-1-sph-10.1177_19417381231171566 – Supplemental material for Comparison of Strength and Power Characteristics Before ACL Rupture and at the End of Rehabilitation Before Return to Sport in Professional Soccer Players [file sj-docx-1-sph-10.1177_19417381231171566.docx]

**ONLINE APPENDIX**

**Table A1** Intra-class correlation coefficients (ICC), coefficient of variation (CV%) and standard error of measurement (SEM) of the performance variables assessed during the bilateral countermovement jump (CMJ) and single leg countermovement jump (SLCMJ)

| **Test** | **Variable** | **CV % (95%CI)** | **ICC (2,1) (95% CI)** | **SEM** |
| --- | --- | --- | --- | --- |
| CMJ | Jump Height | 2.7 (1.6 -3.8) | 0.978 (.922- .994) | 1.4 |
| CMJ | Peak Power Rel | 2.1 (1.2 – 3.0) | 0.966 (.883- .991) | 1.4 |
| CMJ | RSI Mod | 8.6 (5.0 – 12.2) | 0.945 (.875-.976) | 0.0 |
| SLCMJ | Jump Height INV | 5.2 (3.2 – 7.1) | 0.96 (.876- .988) | 1.0 |
| SLCMJ | Peak Power Rel INV | 6.3 (3.9 – 8.7) | 0.781 (.424- .928) | 2.2 |
| SLCMJ | RSI Mod INV | 10.8 (6.6 – 14.9) | 0.907 (.724- .971) | 0.0 |
| SLCMJ | Jump Height UNINV | 5.9 (3.6 – 8.1) | 0.933 (.802-.979) | 1.0 |
| SLCMJ | Peak Power Rel UNINV | 4.0 (2.5 – 5.5) | 0.860 (.612- .955) | 1.4 |
| SLCMJ | RSI Mod UNINV | 8.0 (4.9 – 11.1) | 0.893 (.686- .966) | 0.0 |
| SLCMJ | Jump height symmetry | 4.2 (2.4 - 6.0) | 0.901 (.713- .968) | 4.6 |

INV (involved limb), UNINV (uninvolved limb)

**Table** **A2** Isokinetic and single leg countermovement jump (SLCMJ) results of the uninvolved limb of the injured group and healthy matched controls

| **Test** | **Group 1 Pre-Injury (n=20)** | **Group 1 Post-Injury (n=20)** | **PRE vs POST effect size (95%CI)** | **PRE vs POST P value** | **Pre-Post Percentage difference (95%CI)** | **Group 2: Healthy Controls (n=35)** | **Post-injury vs controls effect size (95%CI)** | **Post-injury vs controls *P* value** |
| --- | --- | --- | --- | --- | --- | --- | --- | --- |
|  | Uninvolved limb | Uninvolved limb |  |  |  |  |  |  |
| **Quad PT Rel (N.m.kg^-1^)** | 3.13±0.44 | 3.39±0.45 | -0.57 (-1.23 to 0.08) | 0.021 | 9.34% (6.45 to 12.23) | 3.06±0.4 | 0.77 (0.19 to 1.36) | **0.018** |
| **HS PT Rel (N.m.kg^-1^)** | 1.79±0.3 | 1.87±0.29 | -0.27 (-0.91 to 0.38) | 0.261 | 7.36% (5.08 to 9.64) | 1.68±0.22 | 0.77 (0.19 to 1.35) | **0.005** |
| **SLCMJ Jump Height (cm)** | 19.2.2±3.4 | 18.6±3.3 | 0.18 (-0.47 to 0.82) | 0.517 | -1.03% (-1.35 to -0.71) | 18.8±2.3 | -0.08 (-0.64 to 0.48) | 0.568 |
| **SLCMJ RSI Mod** | 0.24±0.07 | 0.24±0.06 | -0.03 (-0.67 to 0.61) | 0.900 | 10.7% (7.38 to 14.02) | 0.24±0.05 | 0.10 (-0.46 to 0.66) | 0.987 |
| **SLCMJ Peak Power Rel (W/Kg)** | 32.7±4.4 | 33.0±3.9 | 0.17 (-0.47 to 0.82) | 0.232 | 6.01% (4.15 to 7.87) | 31.9±4.2 | 0.25 (-0.31 to 0.82) | 0.385 |

PT (peak torque), Rel (relative to body mass), N (Newtons), m (meters), kg (kilograms), W (Watts), cm (centimeters)

**Table** **A3** Isokinetic and single leg countermovement jump (SLCMJ) results of the involved limb of the injured group and healthy matched controls

| **Test** | **Group 1 Pre-Injury (n=20)** | **Group 1 Post-Injury (n=20)** | **PRE vs POST effect size (95%CI)** | **PRE vs POST P value** | **Pre-Post Percentage difference (95%CI)** | **Group 2: Healthy Controls (n=35)** | **Post-injury vs controls effect size (95%CI)** | **Post-injury vs controls *P* value** |
| --- | --- | --- | --- | --- | --- | --- | --- | --- |
|  | Involved limb | Involved limb |  |  |  |  |  |  |
| **Quad PT Rel (N.m.kg^-1^)** | 3.2±0.37 | 2.98±0.51 | 0.48 (-0.17 to 1.13) | 0.036 | -7% (-9.2 to -4.8) | 3.06±0.4 | -0.18 (-0.74 to 0.39) | 0.993 |
| **HS PT Rel (N.m.kg^-1^)** | 1.75±0.26 | 1.96±0.19 | -0.90 (-1.58 to -0.23) | **≤0.0001** | 14.2% (9.8 to 18.6) | 1.68±0.22 | 1.32 (0.70 to 1.93) | **≤0.0001** |
| **SLCMJ Jump Height (cm)** | 18.5±4.4 | 14.6±2.9 | 1.03 (0.34 to 1.71) | **0.005** | -12.08% (-16.54 to -9.06) | 18.8±2.3 | -1.64 (-2.28 to -0.99) | **≤0.0001** |
| **SLCMJ RSI Mod** | 0.22±0.08 | 0.18±0.06 | 0.50 (-0.16 to 1.15) | 0.099 | -5.04% (-6.6 to -3.48) | 0.24±0.05 | -0.93 (-1.52 to -0.34) | **0.004** |
| **SLCMJ Peak Power Rel (W/Kg)** | 31.7±4.3 | 30.2±7 | 0.25 (-0.39 to 0.90) | 0.411 | -3.14% (-3.61 to -2.67) | 31.9±4.2 | -0.31 (-0.88 to 0.25) | .325 |

PT (peak torque), Rel (relative to body mass), N (Newtons), m (meters), kg (kilograms), W (Watts), cm (centimeters)

**Figure A1a** Involved limb and **Figure A1b** uninvolved limb single leg countermovement jump (SLCMJ) height pre-injury and post anterior cruciate ligament reconstruction (ACLR). Centimeters (cm). Control group (CTRL)

**Figure A2a** Involved limb and **Figure A2b** uninvolved limb knee extension strength pre-injury and post anterior cruciate ligament reconstruction (ACLR). Newton (N). Meter (m). Kilogram (kg). Control group (CTRL)

**Figure A3a** Involved limb and **Figure A3b** uninvolved limb knee flexion strength pre-injury and post anterior cruciate ligament reconstruction (ACLR). Newton (N). Meter (m). Kilogram (kg). Control group (CTRL)

**Figure A4** Countermovement jump (CMJ) height pre-injury and post anterior cruciate ligament reconstruction (ACLR). Centimeters (cm). Control group (CTRL)

**Figure A5** Knee extension and flexion strength, single leg countermovement jump height, RSI and relative peak power. Newton (N). Meter (m). Centimetre (cm). Metre (m). Second (s). Kilogram (kg). Watt (W). RTS (return to sport)
